# Analysis of wet and dry encounters in the Yangtze River Basin based on the Copula function family and research on the probability of floods and droughts occurrence

**DOI:** 10.1371/journal.pone.0327082

**Published:** 2025-07-23

**Authors:** Jinhang Li, Mengdie Zhao, Yuping Han

**Affiliations:** 1 North China University of Water Resources and Electric Power, Zhengzhou, China; 2 Zhejiang University of Water Resources and Electric Power, Hangzhou, China; 3 Zhejiang Key Laboratory of River-Lake Water Network Health Restoration, Hangzhou, China; University 20 Aout 1955 skikda, Algeria, ALGERIA

## Abstract

As the largest river in China, exploring the evolution characteristics of rainfall in the upper, middle, and lower reaches of the Yangtze River and analyzing the frequency of wet and dry encounters play a significant role in flood and drought prevention in the Yangtze River Basin. This study employs the Mann-Kendall test, 5-year moving curve, Pettitt test method, and Morlet wavelet analysis method to analyze and diagnose the trend, abrupt changes, and periodicity of rainfall in the Yangtze River Basin. By fitting the optimal rainfall marginal distribution and Copula joint distribution model, the probability of wet and dry period encounters and their changing patterns in the upper, middle, and lower reaches of the Yangtze River are calculated and analyzed. Based on the results of wet and dry encounters, a Bayesian network model for flood and drought management is constructed and simulated. The study shows that: (1) There is no significant change in the overall rainfall pattern in the Yangtze River Basin, with periodic changes of 14, 33, and 10 years in the upper, middle, and lower reaches respectively, and abrupt change years in 1997, 1986, and 1979. (2) Apart from the middle and lower reaches of the Yangtze River, where the number of encounters between wet and dry years reaches up to 15 times, the frequency of asynchronous and synchronous wet and dry periods is generally evenly distributed across the upper, middle, and lower reaches. (3) Based on Bayesian prior inference and simulation results, the probabilities of simultaneous floods and droughts occurring in the upper, middle, and lower reaches are 53% and 47%, respectively. When the upper, middle, and lower reaches are all in wet or dry years, the probability of floods and droughts in the Yangtze River Basin is 91%. The research results provide a theoretical basis for scientifically grasping the changes in water conditions in the Yangtze River Basin and for flood and drought prevention.

## 1. Introduction

The analysis of rainfall abundance and scarcity encounters within a basin can provide a scientific basis for accurately understanding the changes in the water regime of the Yangtze River, enhancing inter-regional regulation of water resources during periods of abundance and scarcity, and improving the basin’s disaster prevention and mitigation capacity. This has significant implications for the high-quality development of the Yangtze River economic belt [1]. In recent years, extreme rainfall events have become more frequent, with considerable spatial distribution differences. The suddenness, extremeness, and anomaly of disasters such as torrential rains, floods, and droughts have become increasingly evident. Extreme weather and rare hydrological disasters could occur in any basin, potentially leading to alternating drought and flood conditions, highlighting the importance of calculating the probability of rainfall abundance and scarcity encounters for urban flood and drought prevention in a changing environment [2–4].

Despite recent research efforts that have deeply analyzed the evolution characteristics of rainfall in various locations, limited attention has been given to the encounters between abundant and scarce rainfall across different locations and the connection of these encounters with extreme disasters. Zulfaqar analyzed the spatial and temporal patterns of future precipitation distribution in the Johor River Basin in Malaysia using the latest coupled model intercomparison project phase 6 global circulation models [5]. Qiu used daily vertical displacement time series data from 102 GNSS stations in the Paraná River Basin to invert the water storage changes in the basin from 2013 to 2020 [6]. Zuo focused on the Jixian Caichuan main gully watershed and five small watersheds with different vegetation types, monitoring two typical rainfall-runoff processes and their spatiotemporal variations in hydrogen and oxygen isotopes to explore the differences in runoff processes and their sources in small watersheds [7]. Most existing studies focus on the overall rainfall analysis of a single region, with less attention to the encounters of abundance and scarcity in the upper, middle, and lower reaches within a basin.

The Copula function, capable of constructing multidimensional joint distributions through marginal distributions and correlation structures, reflects the characteristics of dependency among variables, making it an effective tool for linking multiple variable marginal distributions in a joint distribution function. This reflects the stochasticity and dependency features of hydrological events. Shi constructed various marginal and Copula joint distribution models to calculate and analyze the probability of encounters between abundance and scarcity of natural runoff and the Three Gorges Project and their changing patterns [8]. Du applied the Bayesian network model to simulate the ecological safety early warning risks under different scenarios, providing insights for the maintenance of the safety and sustainable development of river basins’ ecosystems [9]. Zhou proposes a framework for constructing rainfall and its joint distribution of HHT and HLT using the Copula function to determine the most likely HHT (HHT-M) and HLT (HLT-M) values associated with rainfall at different recurrence periods [10].

However, these studies have obvious limitations: on the spatial scale, they mostly focus on local river basins and lack systematic analysis of complex river basins like the Yangtze River; At the mechanism level, the causal relationship between the statistical characteristics of abundance and scarcity encounters and actual drought and flood disasters has not been established, and the existing models lack physical process coupling. Methodologically, it is mostly limited to two-dimensional analysis and it is difficult to depict the three-dimensional rainfall co-variation in the upper, middle and lower reaches. In particular, the current research has not yet addressed three key issues: How to quantify the compound effects of cross-regional rainfall encounters? What quantitative relationship exists between multi-dimensional rainfall anomalies and drought and flood in river basins? How to organically combine statistical models with disaster-causing physical mechanisms? These gaps have severely restricted the improvement of the comprehensive risk prevention and control capacity of large-scale river basins. Current research lacks analysis of abundance and scarcity encounters in the Yangtze River Basin and the relationship between rainfall encounters and floods and droughts. It is unclear whether there is a correlation between the evolution characteristics of rainfall in the upper, middle, and lower reaches and the encounters of abundance and scarcity, and if so, to what extent. Especially, the extent to which the encounters of abundance and scarcity in the upper, middle, and lower reaches may be related to droughts and floods remains unknown. To fill this gap, this study uses the Mann-Kendall trend test, Pettitt change point test, and Morlet wavelet analysis to diagnose and analyze the long-term trends, abrupt changes, and periodicities of rainfall during the flood season, dry season, and throughout the year in the upper, middle, and lower reaches of the Yangtze River. Five two-dimensional and three three-dimensional Copula functions are selected to construct the joint distribution function of rainfall during the flood season, dry season, and throughout the year between the upper, middle, and lower reaches, calculating the frequency of abundance and scarcity encounters during these periods. Based on these results, a Bayesian network model for flood and drought management is constructed, and simulation models for flood and drought management are conducted. The findings provide a theoretical basis for flood and drought prevention in the Yangtze River Basin and for accurately grasping water regime changes.

## 2. Materials and methods

### 2.1. Study area and data source

The Yangtze River Basin spans across the eastern, central, and western economic zones of China, including 19 provinces, cities, and autonomous regions. It is the third-largest river system globally, covering a total area of 1.8 million square kilometers, which accounts for 18.8% of China’s total land area. The average annual precipitation within the basin is 1067 mm. Due to its vast geographical range and complex terrain, the monsoon climate is very typical, leading to significant spatial and temporal unevenness in annual rainfall distribution. The Tibetan Plateau and the Western Sichuan Plateau within the basin receive relatively less rainfall, while the middle and lower reaches, especially in provinces like Hunan, Jiangxi, and Zhejiang, experience more abundant rainfall. The maximum monthly rainfall in the upper reaches typically occurs in July and August, with these two months accounting for about 40% of the annual total^10^. In contrast, the southern areas of the middle and lower reaches mostly see their highest rainfall in May and June, with these two months contributing to about 35% of the annual total; the northern areas of the middle and lower reaches primarily in June and July, with these two months accounting for about 30% of the annual total^11^.

Based on the analysis of the rainfall patterns and causes in the Yangtze River, this study divides the calculation period into three-time scales: the flood season (May to October), the dry season (November to April of the following year), and the entire year. A total of 52 meteorological stations were selected, including 29 in the upper reaches, 20 in the middle reaches, and 3 in the lower reaches of the Yangtze River. The ArcGIS software was used to construct Thiessen polygons for the upper, middle, and lower reaches of the Yangtze River, respectively, to calculate the area-averaged rainfall in the upper, middle, and lower reaches.

In this paper, the actual precipitation data of 16 representative stations in the Yangtze River Basin from 1960 to 2020 were collected and sorted from China Meteorological Data Network (http://data.cma.cn/). The precipitation data adopted in this study mainly include the daily observation data of 756 national-level ground meteorological stations provided by the China Meteorological Administration (from 1951 to 2022). Through multi-source data fusion and strict quality control processes, a high-quality dataset suitable for the analysis of abundance and scarcity in the Yangtze River Basin was constructed. During the data collection stage, the observation data of the stations are from the China Meteorological Data Network. In the data processing stage, the threshold method, missing value interpolation and time consistency adjustment are adopted; In the data integration stage, the Tyson polygon method is used to calculate the average precipitation of the basin surface, and the flood season, dry season and annual scale data are divided to meet different analysis requirements.2.2 Rainfall trend analysis

This study utilizes observed rainfall data from the flood season, dry season, and throughout the year for the upper, middle, and lower reaches of the Yangtze River from 1959 to 2020. The Mann-Kendall trend test (hereafter referred to as the “M-K test”) and a 5-year moving average method are employed to analyze the long-term trend of rainfall [11].

### 2.3. Diagnosis of rainfall abrupt change points

There are various methods to analyze changes in time series, including the cumulative anomaly method, M-K abrupt change test, etc. Among these, the Pettitt test for abrupt changes is characterized by its clear physical meaning and simple calculation steps, which can accurately determine the number and location of change points and statistically verify their significance^12^. Therefore, this study adopts the Pettitt test to diagnose the abrupt change points in the annual rainfall of the Yangtze River Basin and uses the M-K abrupt change test for result comparison to ensure the accuracy of the diagnosed change points [12,13].

The abrupt change test statistic $U_{t,n}$ defined by the Pettitt test is:


$$U_{t,n}=\sum_{i=1}^{t}\sum_{j=t+1}^{n}\text{sgn}\left(x_{j}-x_{i}\right)\;1\leq t\leq n$$ (1)

If the moment τ satisfies:


$$K_{\tau }=\left|U_{\tau ,n}\right|=\max\left|U_{t,n}\right|$$ (2)

$$p=2\exp\left(\frac{-6K_{\tau }^{2}}{T^{2}+T^{3}}\right)$$ (3)

If p ≤ 0.05, it indicates that the detected change point τ is statistically significant.

### 2.4. Analysis of rainfall periodicity

When analyzing the periodicity of rainfall, signals often exhibit non-stationarity. Morlet wavelet analysis, as a time-frequency analysis method, provides an effective means to extract the local characteristics of these non-stationary signals. Because of its unique time-frequency localization ability, Morlet wavelet analysis method has shown significant advantages in the analysis of rainfall periodicity in the Yangtze River Basin: It can effectively process non-stationary signals, identify key periodicity laws such as 14 years in the upper reaches and 33 years in the middle reaches through multi-scale decomposition, and visually present the spatio-temporal evolution of the periodicity intensity with the help of time-frequency energy spectra, overcoming the limitations of traditional Fourier analysis on dynamic signal analysis. At the same time, its flexible wavelet basis function parameter adjustment function can adapt to complex rainfall patterns. However, this method is limited by boundary effect, subjectivity of parameter selection and noise sensitivity, which needs to be optimized by data extension, statistical testing and preprocessing. Nevertheless, in the face of the non-stationary characteristics of rainfall caused by the superposition of climate change and human activities, Morlet wavelet is still an indispensable tool. Its multi-time scale analytical ability can not only reveal the correlation between climate driving mechanism such as ENSO and rainfall cycle, but also provide scientific basis for medium – and long-term early warning of floods and droughts in the Yangtze River Basin and intelligent reservoir operation. It highlights the irreplaceability of this method in dynamic hydrological signal analysis and disaster prevention and control decision support [14,15].

In this paper, Morlet wavelet analysis is employed to study the periodic variations in rainfall in the upper, middle, and lower reaches of the Yangtze River.

### 2.5. Analysis of wet and dry encounters in the upper, middle, and lower reaches of the Yangtze River

This study uses a quintile method to classify the levels of abundance and scarcity in the Yangtze River Basin and constructs a joint distribution function for the upper, middle, and lower reaches using Copula functions. Based on the levels of abundance and scarcity and the optimal Copula joint distribution function, the frequency of wet and dry encounters in the upper, middle, and lower reaches of the Yangtze River is calculated.

#### 2.5.1. Criteria for classification of wet and dry levels.

This study adopts a quintile method, classifying the frequency of abundant and scarce rainfall into five levels: wet period (P < 12.5%), slightly wet period (P < 12.5%), normal period (37.5% ≤ P < 62.5%), slightly dry period (62.5% ≤ P < 87.5%), and dry period (P > 87.5%) for the flood season, non-flood season, and throughout the year in the upper, middle, and lower reaches of the Yangtze River. The specific classification results are shown in Table 1.

**Table 1:** Classification Standards for Wet and Dry Levels (Rainfall/ mm).

Principle of division	Upstream			Midstream			Downstream		
	Flood period	Dry period	Year round	Flood period	Dry period	Year round	Flood period	Dry period	Year round
Rich (P < 12.5%)	>692.0	>168.3	>844.7	>1128.4	>631.3	>1660.0	>987.0	>512.7	>1457.3
Slightly rich (12.5% ≤ P < 37.5%)	(638.9, 692.0)	(138.4, 168.3)	(770.4, 844.7)	(915.5, 1128.4)	(539.3, 631.3)	(1451.0, 1660.0)	(818.0, 987.0)	(425.0, 512.7)	(1213.6, 1457.3)
Normal (37.5% ≤ P < 62.5%)	(584.3, 638.9)	(124.1, 138.4)	(714.6, 770.4)	(737.7, 915.5)	(474.7, 539.3)	(1262.8, 1451.0)	(707.9, 818.0)	(365.6, 425.0)	(1121.9, 1213.6)
Slightly poor (62.5% ≤ P < 87.5%)	(494.2, 584.3)	(102.1, 124.1)	(630.9, 714.6)	(595.7, 737.7)	(378.4, 474.7)	(1077.3, 1262.8)	(585.4, 707.9)	(296.0, 365.6)	(958.8, 1121.9)
Poor (P > 87.5%)	<494.2	<102.1	<630.9	<595.7	<378.4	<1077.3	<585.4	<296.0	<958.8

#### 2.5.2. Copula function parameter estimation and goodness-of-fit test.

In the research on constructing the joint distribution model for rainfall in the upper, middle, and lower reaches of the Yangtze River, the initial step involved fitting the marginal distribution functions. This study selected five types of marginal distribution functions: P-III distribution, Log-Normal (LOGN), Exponential (EXP), Generalized Extreme Value (GEV), and Gumbel distributions, to perform a detailed fitting of the marginal distributions of rainfall across the upper, middle, and lower reaches of the Yangtze Rive [16]. The parameters of these marginal distributions were estimated using the method of moments, and the goodness of fit between the theoretical and empirical distributions was verified using the Kolmogorov-Smirnov test (hereinafter referred to as “K-S test”) and the Anderson-Darling test (hereinafter referred to as “A-D test”). The optimal marginal distribution was determined through evaluation methods such as the Root Mean Square Error (RMSE)^17^, Mean Absolute Error (MAE)^17^, Akaike Information Criterion (AIC), and Bayesian Information Criterion (BIC), aiming to enhance the accuracy and reliability of the joint distribution model for rainfall in the Yangtze River Basin [17].

This study selected five common bivariate Copula functions: Gaussian Copula, Frank Copula, Clayton Copula, Gumbel Copula, and t Copula, to construct the joint distribution functions of rainfall between the upper, middle, and lower reaches during the flood season, dry season, and throughout the year. The parameters of bivariate Copula functions were calculated using a non-parametric method, thus obtaining expressions for Kendall’s τ and the Copula parameter θ [18]. In terms of the goodness-of-fit test, the study employed criteria such as K-S, Ordinary Least Squares (OLS), and RMSE minimum criterion to assess the fit of the five bivariate Copula functions, thereby selecting the optimal Copula function [19].

When constructing the triradiate Copula function for rainfall in the upper, middle, and lower reaches during the flood season, dry season, and throughout the year, this study selected three triradiate Copula functions: Gumbel-Hougaard Copula, Clayton Copula, and Frank Copula. Parameters were estimated using the maximum likelihood estimation method. The goodness-of-fit was evaluated using criteria such as K-S, OLS, and RMSE minimum criterion, to select the optimal Copula function [20]. The formula and parameters of the distribution function to be selected are shown in Table 2.

**Table 2:** Formulas for six Copula functions and explanation of parameters.

Copula	Formulas	Parameters
Gumbel	$\begin{array}{c} {}*20cC(U,V)=\exp\left\{-{\left[{\left(-\ln U\right)}^{\rho }+{\left(-\ln V\right)}^{\rho }\right]}^{1/\rho }\right\}\\ C(U,V,Z)=\exp\left\{-{\left[{\left(-\ln U\right)}^{\rho }+{\left(-\ln V\right)}^{\rho }+{\left(-\ln Z\right)}^{\rho }-2\right]}^{1/\rho }\right\} \end{array}$	ρ≥1
Clayton	$\begin{array}{c} {}*20cC(U,V)={\left(U^{-\rho }+V^{-\rho }-1\right)}^{-1/\rho }\\ C(U,V,Z)={\left(U^{-\rho }+V^{-\rho }+Z^{-\rho }-2\right)}^{-1/\rho } \end{array}$	ρ≥0
Frank	$\begin{array}{c} {}*20cC(U,V)=-\frac{1}{\rho }\ln\left(1+\frac{\left(\exp(-\rho U)-1)(\exp(-\rho V)-1\right)}{\left(\exp(-\rho )-1\right)}\right)\\ C(U,V,Z)=-\frac{1}{\rho }\ln\left(1+\frac{\left(\exp(-\rho U)-1)(\exp(-\rho V)-1)(\exp(-\rho Z)-1\right)}{{\left(\exp(-\rho )-1\right)}^{2}}\right) \end{array}$	ρ≠0

### 2.6. Bayesian network model for flood and drought management

A Bayesian network is a directed acyclic graph structure based on conditional probabilities and Bayes’ theorem. The Bayesian network model for flood management combines Bayesian statistics with grid methods to assess and manage the probability of flood occurrence. This model accounts for uncertainties and dependencies among variables. Its dynamic updating feature enhances the flexibility and adaptability of flood management, allowing for timely responses to environmental changes [21,22]. The main steps in Bayesian network modeling include: (1) Determining the network structure, (2) Obtaining network parameters, and (3) Simulating with posterior knowledge.

<V, E> indicates the directed acyclic graph of the network structure. Nodes V={V1, V2,...., V6}. Represents variables, and the directed edge E between nodes represents the correlation between variables. For a directed edge (V_i_, V_j_), V_i_ is called the parent node of V_j_, V_j_ is the child node of V_i_, and a node without a parent is called the root node (V1), and a node without children is called a leaf node (V6). If the parent node set of V_i_ and the non-descendant node set of V_i_ are represented by fa (V_i_) and A(V_i_) respectively, the Bayesian network system contains the following conditional assumptions:

P(V_i_ | fa (V_i_), A (V_i_))= P(V_i_ | fa (V_i_))).

That is, in the case of a given parent, the child node is independent of its non-parent condition.

P represents the system root node probability and non-root node conditional probability. According to the conditional independence hypothesis, the conditional probability distribution of the non-root node can be expressed by P(V_i_ | pa (V_i_)), which expresses the correlation between the node and its parent node.

## 3. Results and analysis

### 3.1 Results of rainfall trend analysis

In this paper, under the significance level of a = 0.05, the Mann-Kendall (M-K) trend analysis was conducted on the rainfall during the flood season, dry season, and throughout the year for the upper, middle, and lower reaches of the Yangtze River from 1959 to 2020. According to the test results, the Z-values for the dry season rainfall in the upper and lower reaches are 2.0895 and 2.4053, respectively, both of which are greater than Za/2 = 1.96. Thus, there is a significant increasing trend in the rainfall during the dry season in the upper and lower reaches of the Yangtze River. The Z-values for the rainfall during the flood season, throughout the year, and the dry season in the middle reaches are positive but all less than Za/2, indicating that the increasing trend in rainfall is not significant. The M-K test results are presented in Table 3.

**Table 3:** Results of the Mann-Kendall test.

M-K trend test results		Z	Trend	Degree of significance
Flood period	Upstream	0.060741	↑	Not significant
	Midstream	0.70459		
	Downstream	1.069		
Dry period	Upstream	2.0895	↑	Significant
	Midstream	0.92326		Not significant
	Downstream	2.4053		Significant
Year round	Upstream	0.81393	↑	Not significant
	Midstream	0.86252		
	Downstream	1.1541		

**Fig 1:**
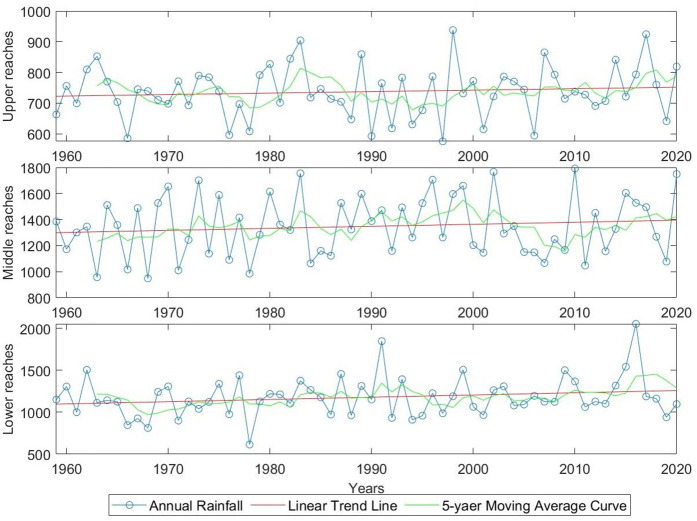
Trends in annual rainfall in the upper, middle, and lower reaches of the Yangtze River, 1959–2020.

The linear trend and 5-year moving average analysis of the annual rainfall in the upper, middle, and lower reaches of the Yangtze River from 1959 to 2020 are shown in Fig 1. The graph indicates that the annual rainfall in the Yangtze River Basin has slightly increased, with the overall rainfall trend remaining stable and showing no significant changes.

### 3.2. Results of rainfall abrupt change point diagnosis

**Fig 2:**
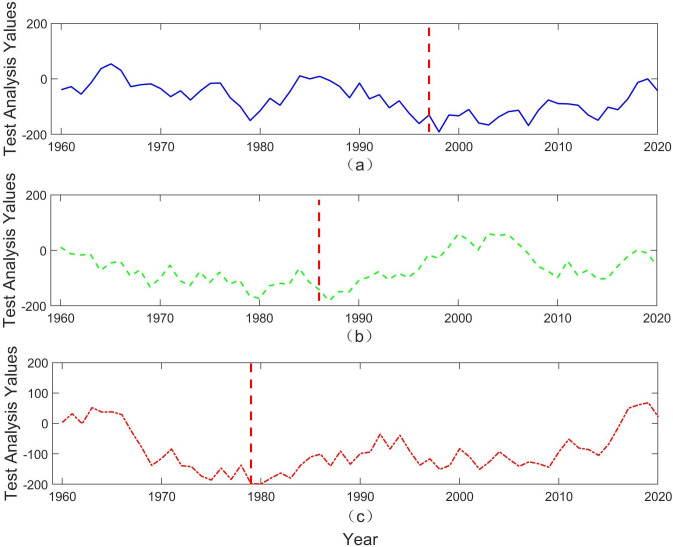
Pettitt Test Results for Annual Rainfall in the Upper, Middle, and Lower Reaches of the Yangtze River.

This study utilized the Pettitt test to analyze the characteristics of abrupt change points in the rainfall patterns of the upper, middle, and lower reaches of the Yangtze River, with the results further verified using the Mann-Kendall (M-K) test. The results of the Pettitt test are illustrated in Fig 2, while the M-K test results are provided in the Supplementary Information. According to Fig 2, at a significance level of 0.05, there is one abrupt change point each in the rainfall series of the upper, middle, and lower reaches of the Yangtze River, occurring in 1997, 1986, and 1979, respectively. In the upper reaches, the mean annual rainfall was 725.7 mm from 1959 to 1997, which increased to 757.1 mm from 1997 to 2020. In the middle reaches, the mean annual rainfall was 1304.3 mm from 1959 to 1986, which increased to 1382.8 mm from 1986 to 2020. In the lower reaches, the mean annual rainfall was 1101.9 mm from 1959 to 1979, which increased to 1213.8 mm from 1979 to 2020.

### 3.3. Results of rainfall periodicity analysis

**Fig 3:**
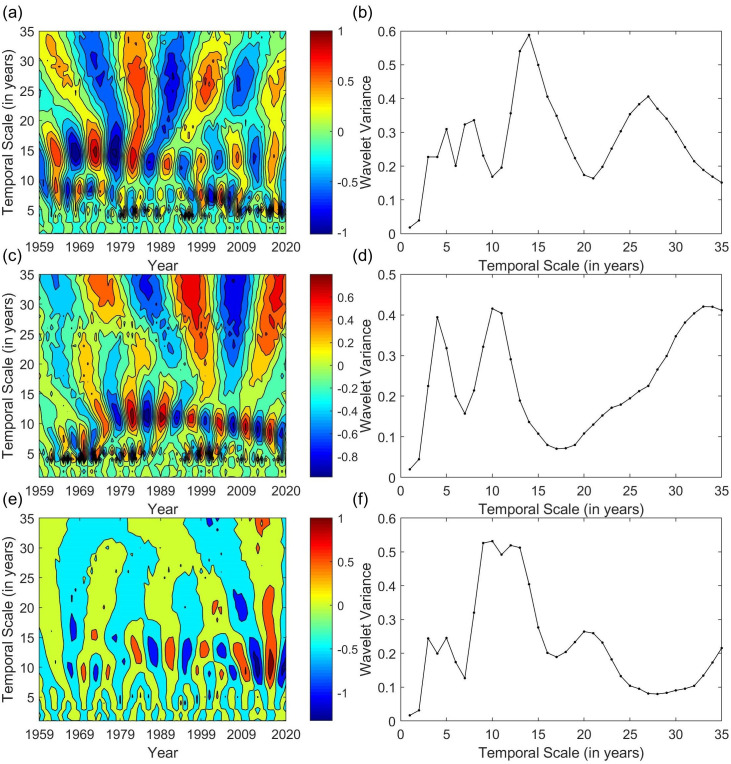
Morlet Wavelet Analysis Results of Annual Rainfall in the Upper, Middle, and Lower Reaches of the Yangtze River.

This study conducted a Morlet wavelet analysis on the periodic variations in the rainfall series of the upper, middle, and lower reaches of the Yangtze River from 1959 to 2020, with the results presented in a graph. According to Fig 3, over the 62-year study period, the annual rainfall in the upper, middle, and lower reaches exhibited periodic changes of 14 years, 33 years, and 10 years, respectively. Specifically, the upper reach showed 25 distinct alternating wet and dry processes, the middle reach showed 33 such processes, and the lower reach showed 28. In the next major cycle, the rainfall in the middle reach is expected to be in a dry period, while the rainfall in both the upper and lower reaches is expected to be in a wet period.

### 3.4. Analysis of wet and dry encounters in the upper, middle, and lower reaches of the Yangtze River

This study, based on the optimally selected Copula functions, established bivariate and triradiate joint distribution models for rainfall in the upper, middle, and lower reaches of the Yangtze River, obtaining the joint distribution frequency of rainfall among these regions. By integrating scientifically established criteria for classifying levels of abundance and scarcity, the study further calculated the frequency of wet and dry encounters in the upper, middle, and lower reaches of the Yangtze River.

#### 3.4.1. Optimal Copula function fitting results.

For the joint distribution model of rainfall between pairs of the upper, middle, and lower reaches, this study constructed and optimally selected among five bivariate Copula functions, including the Gaussian Copula. For the overall joint distribution model of rainfall across the upper, middle, and lower reaches, the study constructed and optimally selected among three Copula functions, including the Gumbel-Hougaard Copula, to choose the optimal Copula joint distribution function.

The study determined the optimal marginal distribution functions using principles of minimal RMSE, MAE, AIC, BIC, and through K-S and A-D tests. The optimal marginal distribution functions and parameter values for rainfall in the upper, middle, and lower reaches of the Yangtze River are presented in Table 4. The numerical results of each test and the curve fitting graphs of empirical frequency versus theoretical frequency are provided in the Supplementary Information.

**Table 4:** Optimal Rainfall Margin Distribution Parameter Values for the Upper, Middle, and Lower Reaches of the Yangtze River.

	Flood period	Dry period	Year round
Upstream	P-III:b = −0.01242, g = 7.8293	P-III:b = −0.20012, g = 5.1517	P-III:b = −0.20012, b = 5.1517
Midstream	LOGN:s = 0.4523, m = 6.0825	GEV:k = −0.357, s = 115.37	P-III: b = −0.00705, g = 11.401
Downstream	LOGN:s = 0.2258, m = 6.7642	P-III:b = −0.01937, g = 8.1859	LOGN: s = 0.17713, m = 7.1365

Based on the optimal marginal distribution function results, this study constructed bivariate and triradiate Copula joint distribution models for the rainfall during the flood season, dry season, and throughout the year in the upper, middle, and lower reaches of the Yangtze River. The chosen Copula joint distribution models were subjected to goodness-of-fit tests using K-S, OLS, and RMSE methods, and the optimal Copula function model was selected based on the test results.

For constructing the bivariate joint distribution models between the upper, middle, and lower reaches of the Yangtze River, this study calculated the correlation parameters using the correlation coefficient method. Based on the principles of minimum K-S, OLS, RMSE, and through K-S and A-D tests, the optimal rainfall Copula joint distribution function was selected from among five bivariate Copula functions, including the Gaussian Copula. The optimal Copula joint distribution function and corresponding parameter values obtained from the goodness-of-fit tests are presented in Table 5, with the goodness-of-fit test results provided in the Supplementary Information.

**Table 5:** Optimal Copula joint distribution function parameters for pairwise comparisons among the Upper, Middle, and Lower reaches of the Yangtze River.

Project		Optimal Copula function	Parameter value
Flood period	Upstream-Midstream	Gumbel Copula	1.30714
	Upstream-Downstream	Clayton Copula	0.6445
	Midstream-Downstream	Gumbel Copula	1.5149
Dry period	Upstream-Midstream	Gaussian Copula	0.57802
	Upstream-Downstream	Gaussian Copula	0.4593
	Midstream-Downstream	t Copula	0.677
Year round	Upstream-Midstream	Gaussian Copula	0.3139
	Upstream-Downstream	Gaussian Copula	0.3623
	Midstream-Downstream	t Copula	0.0604

**Fig 4:**
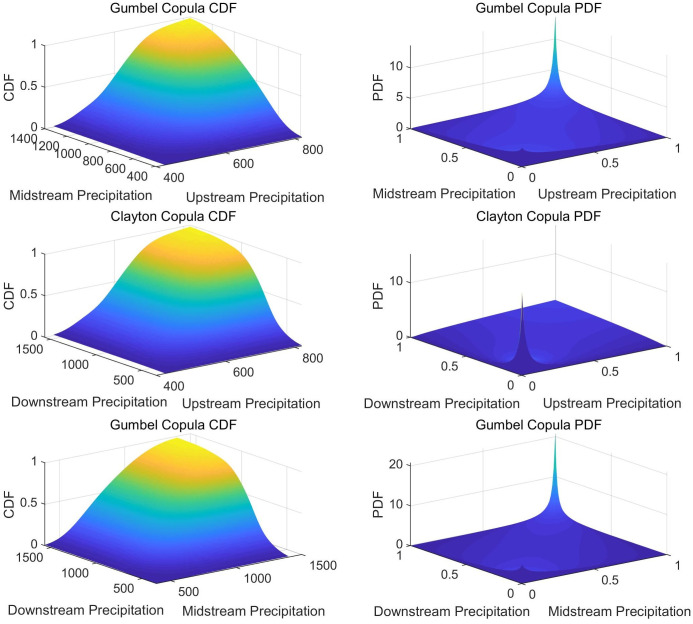
Panels (a), (b), and (c) respectively represent the joint distribution probability graphs and joint probability density function graphs for rainfall during the flood season, dry season, and throughout the year between pairs of the upper, middle, and lower reaches of the Yangtze River.

Fig 4 presents the joint probability distribution and joint probability density function graphs for rainfall during the flood season, dry season, and throughout the year between pairs of the upper, middle, and lower reaches of the Yangtze River. The graphs indicate that as the rainfall in the upper, middle, and lower reaches increases, the joint distribution probability values exhibit a trend of initially slow, then rapid increase, and finally a deceleration in the rate of increase.

For constructing the triradiate joint distribution model among the upper, middle, and lower reaches of the Yangtze River, this study employed the maximum likelihood estimation method to calculate relevant parameter values. Based on the principles of minimum K-S, OLS, RMSE, the optimal rainfall Copula joint distribution function was selected among the Gumbel-Hougaard (GH), Clayton, and Frank triradiate Copula functions.

**Fig 5:**
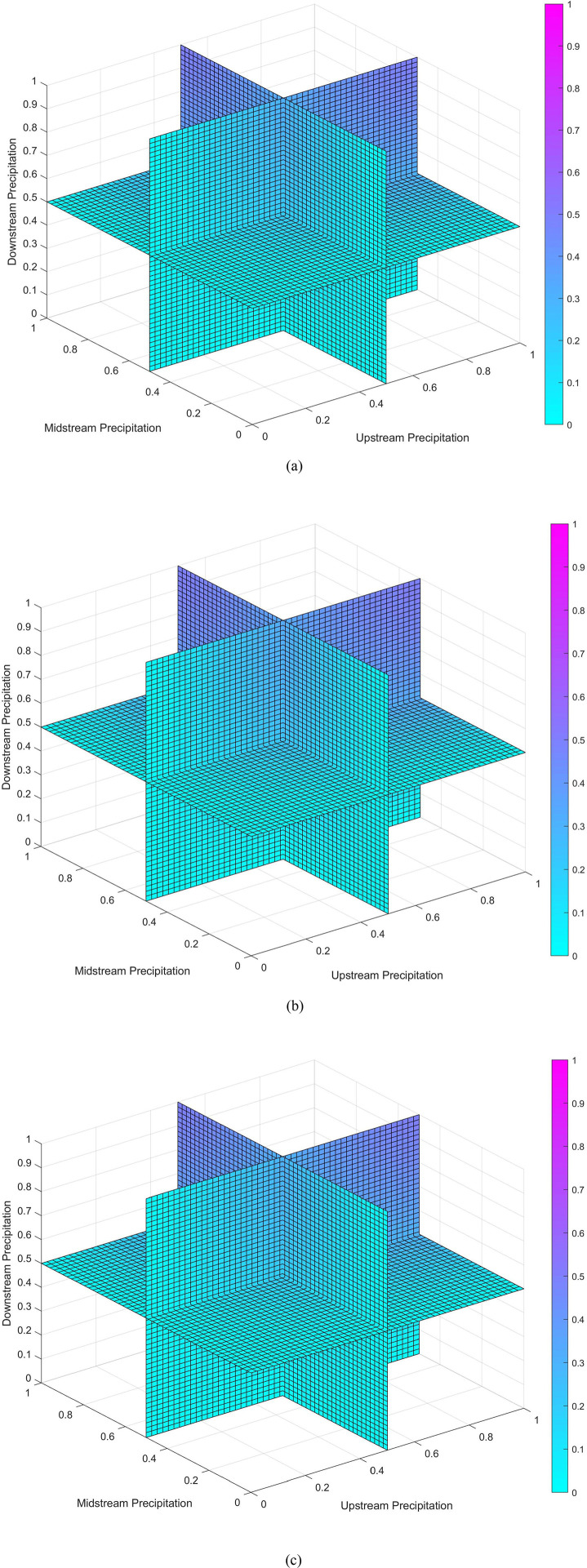
Panels (a), (b), and (c) respectively represent the triradiate joint probability distribution graphs for rainfall during the flood season, dry season, and throughout the year in the upper, middle, and lower reaches of the Yangtze River.

The optimal Copula function for the overall flood season, dry season, and annual rainfall across the upper, middle, and lower reaches of the Yangtze River was the GH Copula, with parameter values of 1.348, 1.522, and 1.318, respectively. Fig 5 represents the triradiate joint distribution probability of rainfall during the flood season, dry season, and throughout the year in the upper, middle, and lower reaches of the Yangtze River. The goodness-of-fit test results for the entire year are provided in the Supplementary Information. The graphs show that as rainfall increases in the upper, middle, and lower reaches of the Yangtze River, the joint probability also gradually increases.

#### 3.4.2. Wet and dry encounters between the upper, middle, and lower reaches of the Yangtze River.

**Fig 6:**
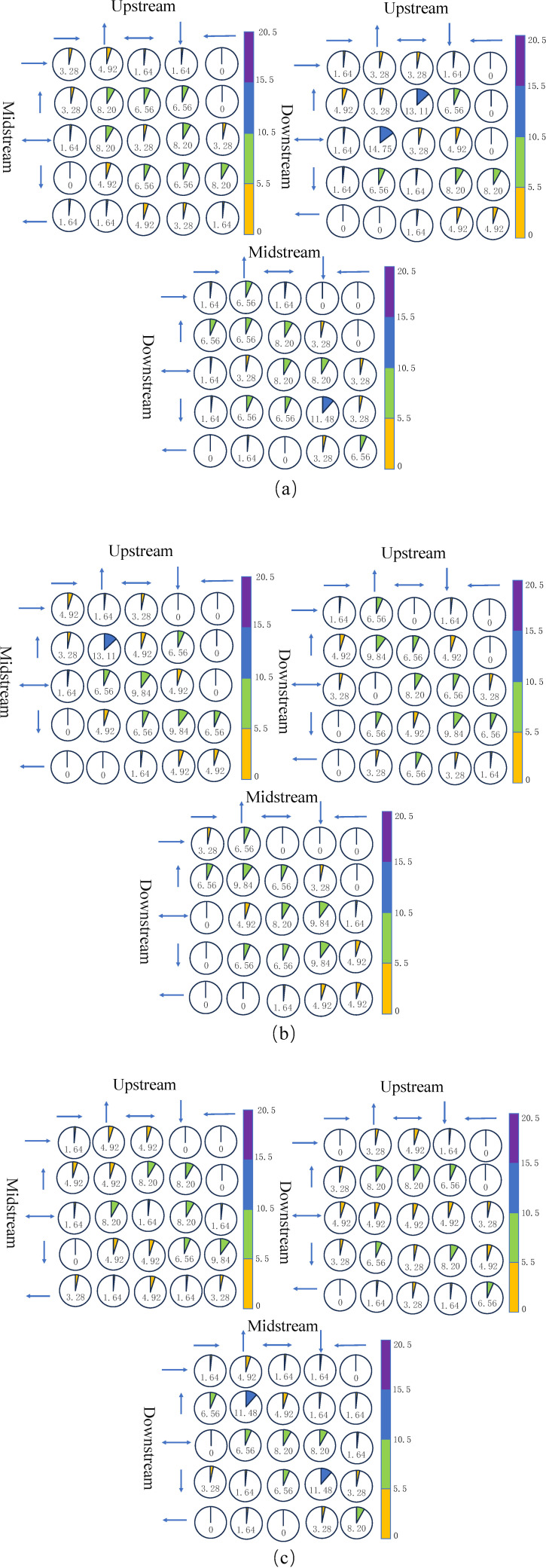
Panels (a), (b), and (c) represent the joint probability graphs for wet and dry encounters during the flood season, dry season, and throughout the year between pairs of the upper, middle, and lower reaches of the Yangtze River. In the graphs, yellow bands indicate encounter probabilities of 0-5.5%, green bands represent probabilities of 5.5-10.5%, blue bands denote probabilities of 10.5-15.5%, and purple bands symbolize probabilities of 15.5-20.5%. The values represent the probabilities of wet and dry encounters between pairs of the upper, middle, and lower reaches.

Based on the division into five levels of abundance and scarcity, there are a total of 25 possible scenarios of encounters between pairs of the upper, middle, and lower reaches of the Yangtze River, including scenarios where both are in a state of abundance, scarcity, or normal conditions. By utilizing the Copula functions for rainfall between pairs of the upper, middle, and lower reaches, the corresponding bivariate joint distributions can be obtained, from which the probabilities of various wet and dry encounters can be calculated. The results of these encounter probabilities are presented in Fig 6.

During the flood season, the highest frequency of encounters between the upper and middle reaches is observed in slightly dry and normal water years, slightly abundant and normal water years, and slightly abundant years with each other, all at 8.20%. Encounters between slightly dry and slightly abundant years, and normal and slightly abundant years, also show a high frequency, each at 6.56%. In contrast, encounters where both the upper and middle reaches are in abundant and dry years are less frequent, at 3.28% and 1.64%, respectively. The upper and lower reaches encounter the highest frequency in slightly abundant and normal water years, reaching 14.75%. High frequencies are also observed in dry and slightly dry years, and slightly dry years with each other, each at 8.20%, with low frequencies for extreme combinations such as abundant and dry years. The middle and lower reaches show the highest frequency of encounters in slightly dry years with each other, at 11.48%, followed by slightly dry and normal water years, at 8.20%. Encounters in normal water years with each other and with slightly abundant years are also high, each at 8.20%. Additionally, combinations of abundant and slightly abundant years are relatively frequent, with encounters in abundant and slightly abundant years, and slightly abundant with abundant years, each at 6.56%. However, it’s noteworthy that encounters in entirely dry years are at a frequency of 6.56%.

During the dry season, the upper and middle reaches most frequently encounter both in slightly abundant years, at 13.11%, with frequencies of both in normal and slightly dry years at 9.84%. Encounters in abundant and dry years are relatively low, with frequencies of both in abundant and dry years at 4.92%. The upper and lower reaches show the highest frequency in slightly abundant and slightly dry years, at 9.84%, with the lowest frequency in entirely abundant and dry years, each at 1.64%. High frequencies are also observed in encounters between slightly abundant and slightly dry years, normal and slightly dry years, and slightly dry and normal water years, each at 6.56%. The middle and lower reaches exhibit the highest frequency in slightly abundant and slightly dry years, each at 9.84%, with a frequency of both in normal water years at 8.20%, and both in dry years at 4.92%, slightly higher than a frequency of 3.28% for entirely dry years. Additionally, encounters between slightly abundant and slightly dry years, and normal and slightly dry years, are also high, at 6.56% each.

Throughout the year, the highest frequency of encounters between the upper and middle reaches in dry and slightly dry years is 9.84%, with frequencies in slightly abundant and normal water years, slightly dry and normal water years, and normal and slightly abundant years each at 8.20%. Frequencies of both in abundant and dry years for the upper and middle reaches are 1.64% and 3.28%, respectively. The upper and lower reaches show a higher frequency in slightly abundant and slightly dry years, each at 8.20%, with no encounters in abundant and dry years, and slightly abundant and dry years. The encounter situation between the middle and lower reaches is similar to that between the upper and lower reaches, with frequencies in slightly abundant and slightly dry years at 11.48%, and a high frequency in entirely dry years at 8.20%. The lowest frequency, at only 1.64%, occurs in scenarios not specified for clarity.

#### 3.4.3. Wet and dry encounters among the upper, middle, and lower reaches of the Yangtze River as a whole.

**Fig 7:**
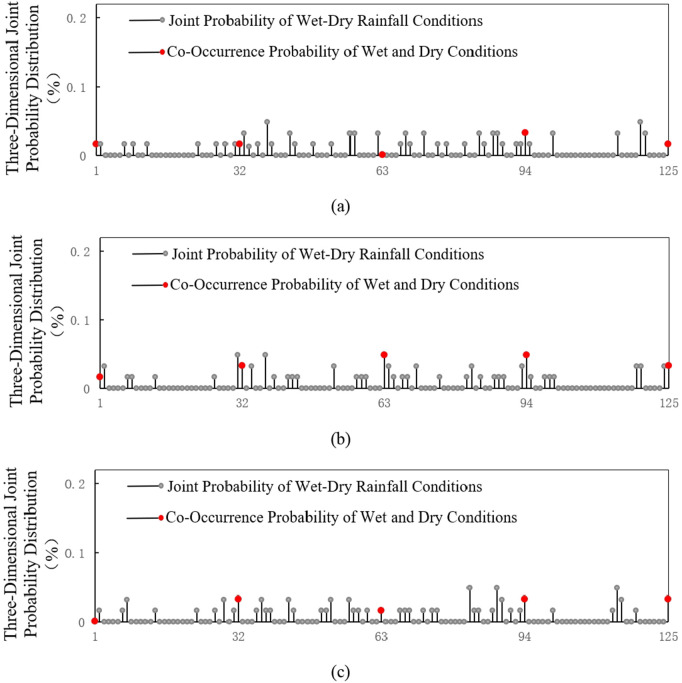
Panels (a), (b), and (c) respectively represent the probability graphs of wet and dry encounters during the flood season, dry season, and throughout the year among the upper, middle, and lower reaches of the Yangtze River.

Based on the classification of abundance and scarcity levels, encounters of wet and dry conditions among the upper, middle, and lower reaches of the Yangtze River as a whole can be divided into 125 scenarios under two situations: synchronous and asynchronous wet and dry encounters. Utilizing the optimally selected GH Copula, a triradiate joint distribution model of rainfall among the upper, middle, and lower reaches of the Yangtze River was established to calculate the probabilities of wet and dry encounters. The results of these encounter probabilities are presented in Fig 7.

The results shown in the graphs indicate that the distribution of wet and dry encounters among the upper, middle, and lower reaches of the Yangtze River as a whole is balanced. Both synchronous and asynchronous wet and dry encounter frequencies range between 1–3 times, with encounter rates between 1.61% and 4.84%, avoiding any situation of excessively high or low frequencies of encounters.

### 3.5 Bayesian network model for flood and drought management based on wet and wry encounters

This study reclassifies slightly abundant and slightly scarce years as abundant and scarce years, respectively, and constructs a Bayesian network model for flood and drought management based on the results of wet and dry encounters, enabling the simulation of floods and droughts in the Yangtze River Basin. The Bayesian grid results are shown in Figure A5-A7 in Supplementary Information.

#### 3.5.1. Prior inference of the Bayesian network model for flood and drought management.

The probability of flooding in the upper and middle reaches of the Yangtze River is 48%, lower than the probability of drought. The probabilities of flooding and drought in the middle and lower reaches are both 50%, while the disparity in probabilities of flooding and drought is more significant in the middle and lower reaches, at 64% and 36%, respectively. The probabilities of simultaneous flooding and drought occurring in the upper, middle, and lower reaches of the Yangtze River are 53% and 47%, respectively.

#### 3.5.2. Simulation of the Bayesian network model for flood and drought management.

In the simulation of wet years, this study constructed flood and drought management models for scenarios where the upper, middle, and lower reaches are all experiencing wet years, as well as scenarios where the upper reach is experiencing a normal year, while the middle and lower reaches are experiencing wet years. When the upper, middle, and lower reaches are all experiencing wet years, the probability of flooding occurring in the upper, middle, and lower reaches is 91%. The probabilities of flooding occurring in the upper and middle reaches, and in the upper and lower reaches, are 52% and 56% respectively, both higher than the probability of drought. Meanwhile, the probability of flooding in the middle and lower reaches is 70%, significantly higher than the 30% probability of drought. When the upper reach is experiencing a normal year, and the middle and lower reaches are experiencing wet years, the probability of both flooding and drought occurring in the upper, middle, and lower reaches is 50%. The probabilities of flooding and drought occurring in the upper and middle reaches, and in the upper and lower reaches, are also 50%, while the probability of flooding in the middle and lower reaches is 70%, significantly higher than the 30% probability of drought.

In the simulation of dry years, this study developed flood and drought management models for scenarios where the upper, middle, and lower reaches are all experiencing dry years, as well as scenarios where the upper reach is experiencing a dry year while the middle and lower reaches are experiencing normal years. When the upper, middle, and lower reaches are all experiencing dry years, the probability of drought occurring in these areas is 91%. The probabilities of drought occurring in the upper and middle reaches, and in the upper and lower reaches, are 69% and 63% respectively, both significantly higher than the 31% and 37% probabilities of flooding. Meanwhile, the probability of flooding in the middle and lower reaches is equal to the probability of drought, both at 50%. When the upper reach is experiencing a dry year, and the middle and lower reaches are experiencing normal years, the probability of both flooding and drought occurring in the upper, middle, and lower reaches is 50%. The probabilities of drought occurring in the upper and middle reaches, and in the upper and lower reaches, are 61% and 58% respectively, both significantly higher than the 39% and 42% probabilities of flooding. Meanwhile, the probability of flooding in the middle and lower reaches is equal to the probability of drought, both at 50%.

## 4. Discussion

### 4.1. Analysis of rainfall evolution characteristics

It has been observed that the rainfall trends in the upper, middle, and lower reaches of the Yangtze River all show an increasing tendency, with lower rainfall amounts in the years of abrupt change points. There is a noticeable difference in rainfall amounts before and after the change points, with amounts being lower before and higher after the change points. This indicates that the results of trend analysis and abrupt change point studies are consistent, showing a gradual increase in rainfall across the Yangtze River Basin. Moreover, the periodic analysis suggests that the next major cycle for both the upper and lower reaches will be a wet period. With both the population and economic output of the Yangtze River Basin exceeding 40% of the national total, the region can leverage its economic advantages to promote sponge city initiatives and enhance rainfall detection infrastructure to mitigate urban flooding caused by extreme rainfall events. As China’s principal agricultural production base, the Yangtze River Basin, with high demands for livestock drinking water and seasonal irrigation water for autumn crops, could coordinate the dispatch of dozens of reservoirs in the upper reaches and the Dongting Lake water system to supplement water downstream during droughts, ensuring irrigation water needs during critical growth periods for crops in the middle and lower reaches and the Two Lakes region.

### 4.2. Probability analysis of flood and drought occurrences based on wet and dry encounters

According to the results of wet and dry encounters in the Yangtze River Basin, extreme scenarios of encounters between abundant and scarce years are less frequent among the upper, middle, and lower reaches, and the encounters are evenly distributed (Figs 7 and 8), making the likelihood of simultaneous flood and drought disasters in these areas low. The middle and lower reaches, in particular, experienced up to 15 encounters between abundant and slightly abundant years, while encounters between dry and slightly dry years were only four times, primarily due to the influence of subtropical monsoons and typhoons. This region, one of the most economically developed in China, could use its economic and technological advantages to promote many research focuses on the impact of climate change and human activities on extreme weather, though determining which driving force is more significant is not straightforward [23–25]. For instance, Xufound that the timing of floods correlates positively with rainfall amounts and peak soil moisture levels, especially with the annual maximum rainfall, suggesting that flood and drought risks in the basin might also be related to the encounters of wet and dry conditions between the upper, middle, and lower reaches [26–28].

Simulation results for abundant years indicate that the probability of flooding in the middle and lower reaches, when both are abundant, is 70%, highlighting the need for attention to flood disasters triggered by extreme rainfall, with a smaller probability for droughts. The encounter situations for rainfall abundance and scarcity between the upper and middle reaches, and the upper and lower reaches, are balanced, with similar probabilities for simultaneous flood and drought disasters. Since rainfall amounts in the middle and lower reaches are higher than in the upper reaches, these areas could store water during the rainy season with higher rainfall and transfer water from the upper reaches during the dry season to avoid the harm caused by extreme disasters. Overall, the distribution of wet and dry conditions across the entire Yangtze River Basin is even, and prior inference results show consistent probabilities for flood and drought disasters. However, simulation results indicate that when the upper, middle, and lower reaches are all abundant, the probability of flood disasters in the Yangtze River Basin reaches 91%, suggesting the importance of real-time rainfall monitoring during the rainy season. Given that mountainous and hilly areas comprise 67.2% of the basin’s total area, the Yangtze River Basin should also implement measures for monitoring landslides to minimize the harm extreme rainfall might cause to people’s lives and property.

The temporal span and spatial resolution of the historical rainfall data in this study may have influenced the validation of long-term cycles (e.g., the 33-year cycle in the middle reaches) and the detection of abrupt change points. Additionally, the study did not systematically incorporate the driving mechanisms of climatic events such as El Niño on wet-dry encounters. Furthermore, simplifications in the assumptions of the Copula model and Bayesian network—particularly in capturing nonlinear dependencies between variables and setting prior probabilities—might introduce biases. Moreover, the exclusion of human interventions (e.g., the Three Gorges Project) in hydrological processes may lead to discrepancies between risk assessments and real-world scenarios. To address these limitations, future work will focus on two key improvements: (1) integrating climate models with human activity data (e.g., water conservancy project operation records) to analyze the synergistic effects of natural and anthropogenic factors on flood-drought risks, and (2) validating the predictive performance of the Bayesian network through historical disaster case studies and real-time monitoring data, thereby establishing a dynamic water resource scheduling framework to support intelligent decision-making.

## 5. Conclusions

This paper employs the Mann-Kendall trend test and 5-year moving average curve to analyze the trend of rainfall in the Yangtze River Basin from 1959 to 2020. Five types of Copula functions, including the Frank Copula, are chosen to fit the joint distribution of rainfall between the upper, middle, and lower reaches during the flood season, dry season, and throughout the year. Three types of Copula functions, including the GH Copula, are selected to fit the joint distribution among the upper, middle, and lower reaches. The optimal Copula joint distribution function is determined through tests such as the Ordinary Least Squares test. The best multidimensional Copula model, combined with a quintile method for dividing rainfall abundance and scarcity thresholds, is used to calculate the probability of wet and dry period encounters under different combination conditions in the upper, middle, and lower reaches during the flood season, dry season, and throughout the year. Based on these results, a Bayesian network model for flood and drought management is constructed and simulated, yielding the following conclusions:

1. Over a 62-year study period, the overall rainfall trend in the Yangtze River Basin is stable with no significant changes. There are periodic changes of 14, 33, and 10 years in the annual rainfall of the upper, middle, and lower reaches, respectively. The upper reach showed 25 significant alternating wet and dry processes, the middle reach had 33, and the lower reach had 28. The years of abrupt rainfall changes in the upper, middle, and lower reaches were 1997, 1986, and 1979, respectively.
2. The probabilities of asynchronous and synchronous wet and dry periods during the flood season, dry season, and throughout the year between the upper, middle, and lower reaches and overall are highly consistent, with a low frequency of extreme rainfall encounters. The probability of encountering wet and above-normal years is higher in the middle and lower reaches. According to the Bayesian network flood management prior inference model, the probability of flooding in the Yangtze River Basin is 53%, slightly higher than the 47% probability of drought.
3. According to the wet year simulation model, when the upper, middle, and lower reaches are all in wet years, the probability of flooding in the Yangtze River Basin is 91%. When the upper reach is in a normal water year, and the middle and lower reaches are in wet years, the probability of flooding is 50%. According to the dry year simulation model, when the upper, middle, and lower reaches are all in dry years, the probability of drought in the Yangtze River Basin is 91%. When the upper reach is in a dry year, and the middle and lower reaches are in normal water years, the probability of drought is 50%.

## Supporting information

S1 File Results. (DOCX)
