# Supplementary material for: Analysis of wet and dry encounters in the Yangtze River Basin based on the Copula function family and research on the probability of floods and droughts occurrence
Source: PLoS One. 2025 Jul 23;20(7):e0327082. doi: 10.1371/journal.pone.0327082 (PMC12286373; doi:10.1371/journal.pone.0327082)
Supplement: S1 File — (DOCX) [file pone.0327082.s001.docx]

## Supplementary Information


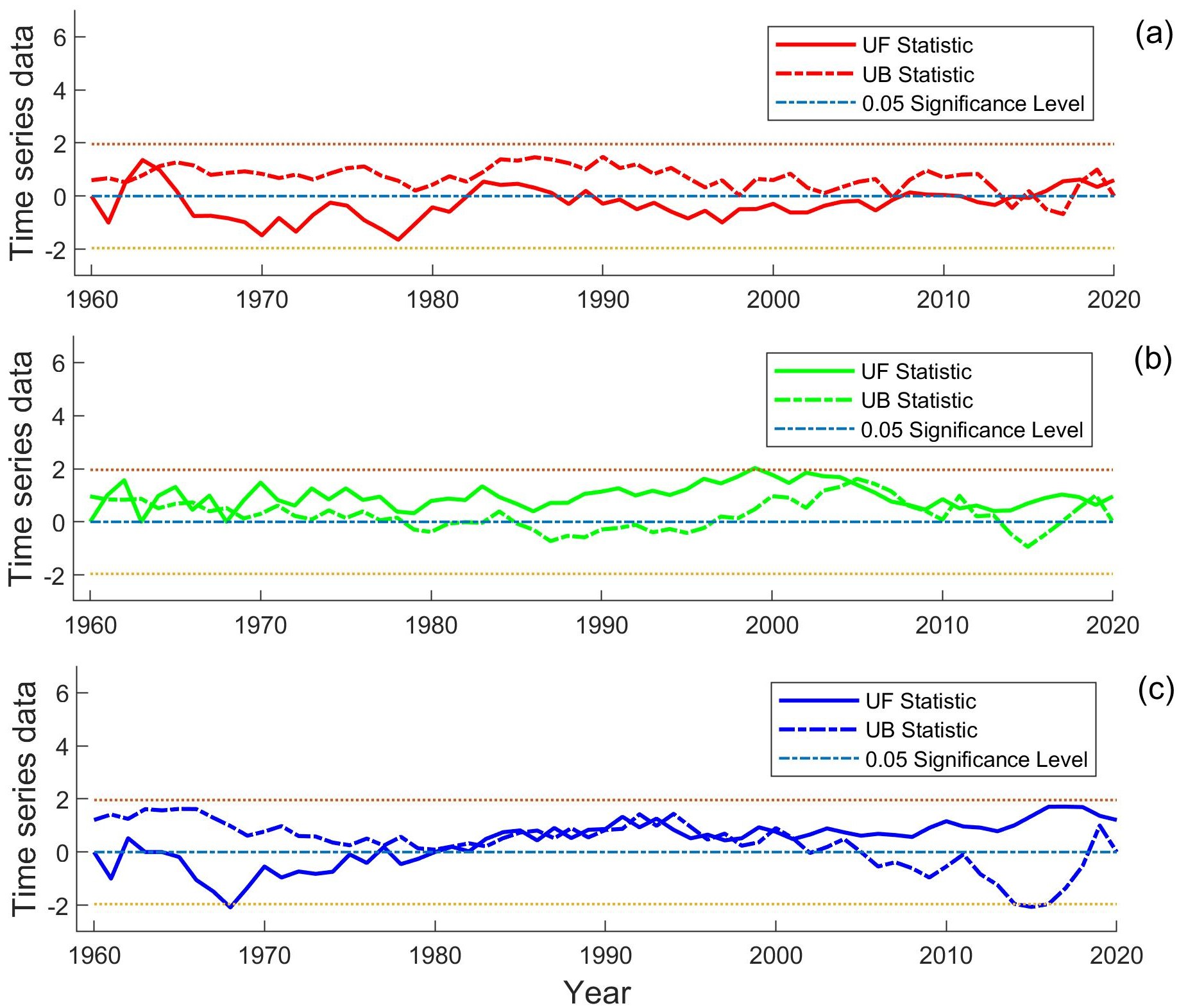


Fig A1. Mann-Kendall trend change point test diagram.

Table A1. Optimal edge distribution function fitting for preferred outcomes.

| Basin | PDF | Year round | | | | Flood period | | | | Dry period | | | |
| --- | --- | --- | --- | --- | --- | --- | --- | --- | --- | --- | --- | --- | --- |
|  |  | RMSE | MAE | AIC | BIC | RMSE | MAE | AIC | BIC | RMSE | MAE | AIC | BIC |
| Upstream | P-Ⅲ | 606.56 | 601.23 | -5.9 | 731.2 | 133.258 | 130.25 | -5.6 | 602.2 | 739.56 | 734.54 | -6.0 | 737.6 |
|  | LOGN | 609.45 | 604.33 | -7.7 | 726.2 | 133.364 | 130.36 | -7.4 | 611.2 | 742.36 | 737.45 | -7.8 | 732.4 |
|  | EXP | 609.45 | 604.52 | -10.3 | 922.3 | 133.256 | 130.58 | -9.8 | 732.6 | 742.58 | 737.56 | -10.3 | 946.9 |
|  | GEV | 609.48 | 604.87 | -5.8 | 730.6 | 133.336 | 130.14 | -5.4 | 605.2 | 742.27 | 737.75 | -5.8 | 735.9 |
|  | Gumbel | 609.45 | 604.54 | -7.8 | 739.5 | 133.448 | 130.47 | -7.4 | 614.3 | 742.11 | 737.65 | -7.8 | 743.4 |
| Midstream | P-Ⅲ | 863.36 | 837.19 | -6.2 | 848.7 | 515.258 | 503.25 | -6.0 | 766.6 | 1360.23 | 1342.3 | -6.2 | 860.1 |
|  | LOGN | 863.36 | 837.23 | -8.1 | 841.2 | 515.742 | 503.86 | -7.9 | 781.8 | 1366.25 | 1347.7 | -8.1 | 856.8 |
|  | EXP | 863.36 | 837.26 | -10.3 | 962.4 | 515.158 | 503.74 | -10.2 | 899.5 | 1366.14 | 1347.4 | -10.4 | 1021 |
|  | GEV | 863.36 | 837.55 | -6.1 | 845.6 | 515.155 | 503.22 | -5.9 | 770.4 | 1366.22 | 1347.1 | -6.1 | 859.7 |
|  | Gumbel | 863.36 | 837.28 | -8.1 | 861.9 | 515.236 | 503.55 | -7.9 | 770.7 | 1366.58 | 1347.3 | -8.1 | 865.9 |
| Downstream | P-Ⅲ | 807.45 | 779.36 | -6.2 | 851.8 | 400.224 | 393.86 | -5.9 | 728.2 | 1200.36 | 1177.5 | -6.3 | 870.2 |
|  | LOGN | 807.56 | 779.25 | -8.1 | 838.7 | 402.367 | 394.91 | -7.8 | 726.3 | 1200.33 | 1177.6 | -8.1 | 854.6 |
|  | EXP | 807.56 | 779.33 | -10.3 | 953.3 | 402.775 | 394.55 | -10.1 | 869.5 | 1200.27 | 1177.7 | -10.4 | 1004 |
|  | GEV | 807.85 | 779.44 | -6.1 | 842.3 | 402.882 | 394.41 | -5.8 | 729.5 | 1200.39 | 1177.1 | -6.1 | 859.1 |
|  | Gumbel | 807.21 | 779.75 | -8.2 | 878.2 | 402.964 | 394.77 | -7.8 | 736.9 | 1200.88 | 1177.3 | -8.2 | 892.1 |

Table A2. Test for optimal marginal distribution function fitting of preferred results.

| Basin | PDF | Year round | | Flood period | | Dry period | |
| --- | --- | --- | --- | --- | --- | --- | --- |
|  |  | K-S test | A-D test | K-S test | A-D test | K-S test | A-D test |
| Upstream | P-Ⅲ | 0.08733 | 0.48854 | 0.11955 | 16.038 | 0.08617 | 0.37401 |
|  | LOGN | 0.08442 | 0.4816 | 0.06075 | 0.17894 | 0.06333 | 0.29303 |
|  | EXP | 0.52086 | 21.674 | 0.39361 | 14.37 | 0.2996 | 8.0636 |
|  | GEV | 0.08402 | 0.48621 | 0.05618 | 0.28979 | 0.08783 | 0.39504 |
|  | Gumbel | 0.09418 | 0.56182 | 0.0909 | 0.88743 | 0.11212 | 1.8652 |
| Midstream | P-Ⅲ | 0.07194 | 0.55769 | 0.09278 | 12.005 | 0.09047 | 0.45119 |
|  | LOGN | 0.08974 | 0.56024 | 0.05478 | 0.21691 | 0.09657 | 0.57343 |
|  | EXP | 0.14042 | 3.6591 | 0.36925 | 15.342 | 0.20221 | 5.9233 |
|  | GEV | 0.06628 | 0.49111 | 0.04626 | 0.15214 | 0.08378 | 0.35771 |
|  | Gumbel | 0.08488 | 0.72774 | 0.10315 | 1.5573 | 0.11275 | 1.105 |
| Downstream | P-Ⅲ | 0.08406 | 0.44525 | 0.05067 | 0.26325 | 0.07929 | 0.49752 |
|  | LOGN | 0.056 | 0.18502 | 0.05805 | 0.35186 | 0.06465 | 0.25592 |
|  | EXP | 0.31776 | 10.514 | 0.21719 | 5.9241 | 0.34929 | 12.961 |
|  | GEV | 0.08078 | 0.41101 | 0.03954 | 0.21127 | 0.08863 | 0.42938 |
|  | Gumbel | 0.09047 | 0.4848 | 0.09091 | 0.9993 | 0.10199 | 0.51088 |


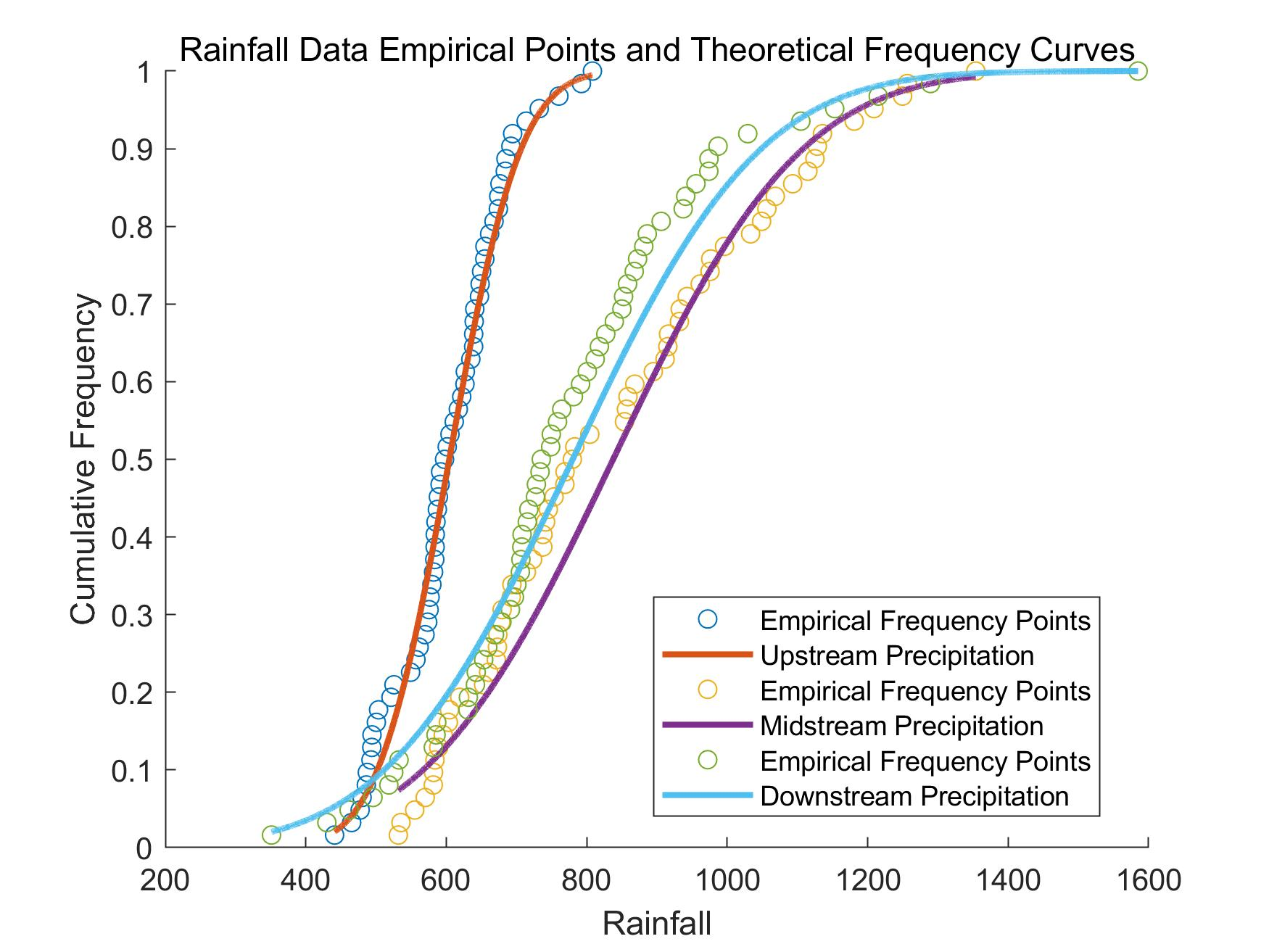


Fig A2. Empirical points and theoretical frequency curves of rainfall data during flood season.


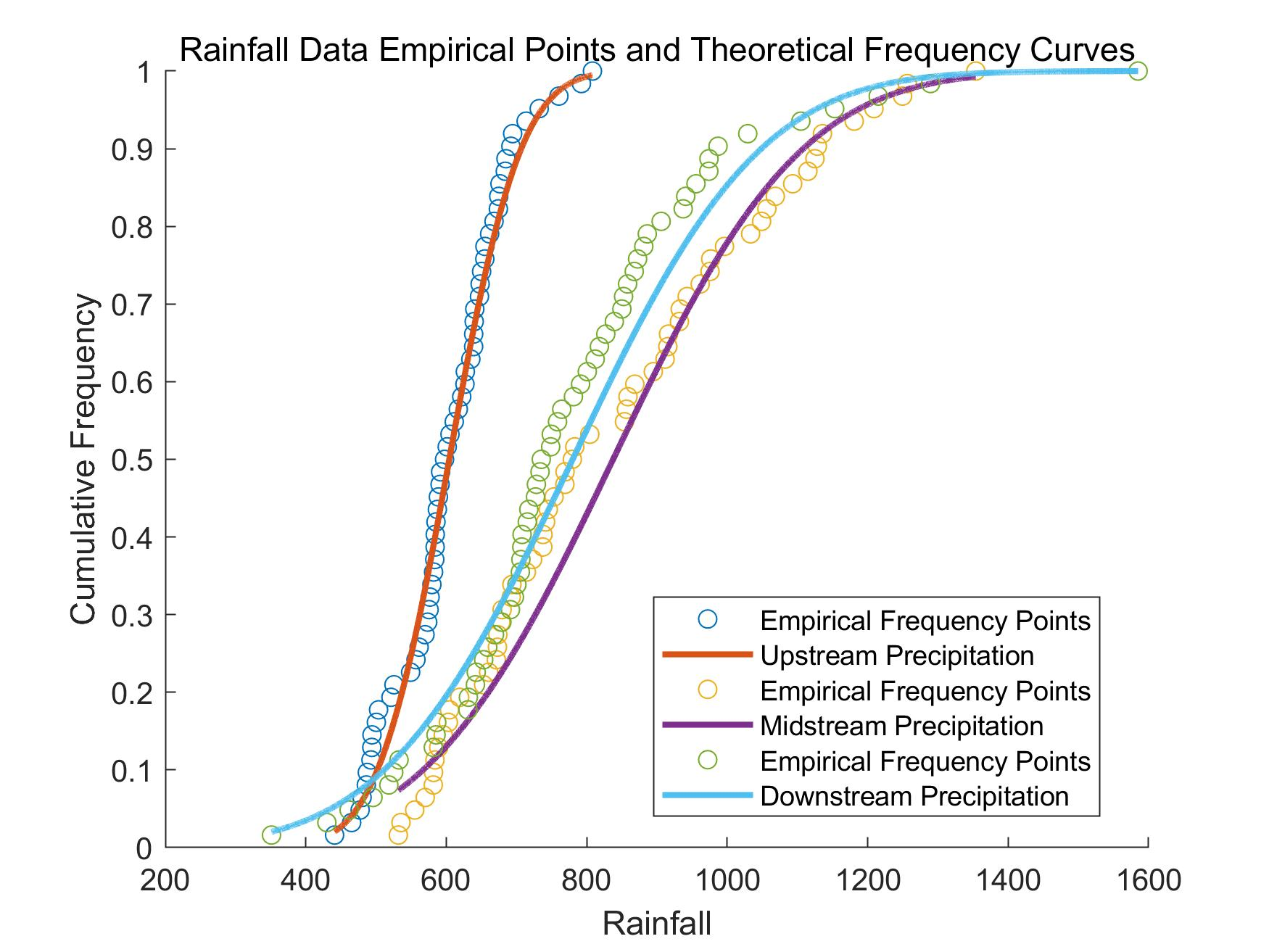


Fig A3. Empirical points and theoretical frequency curves of rainfall data during dry season.


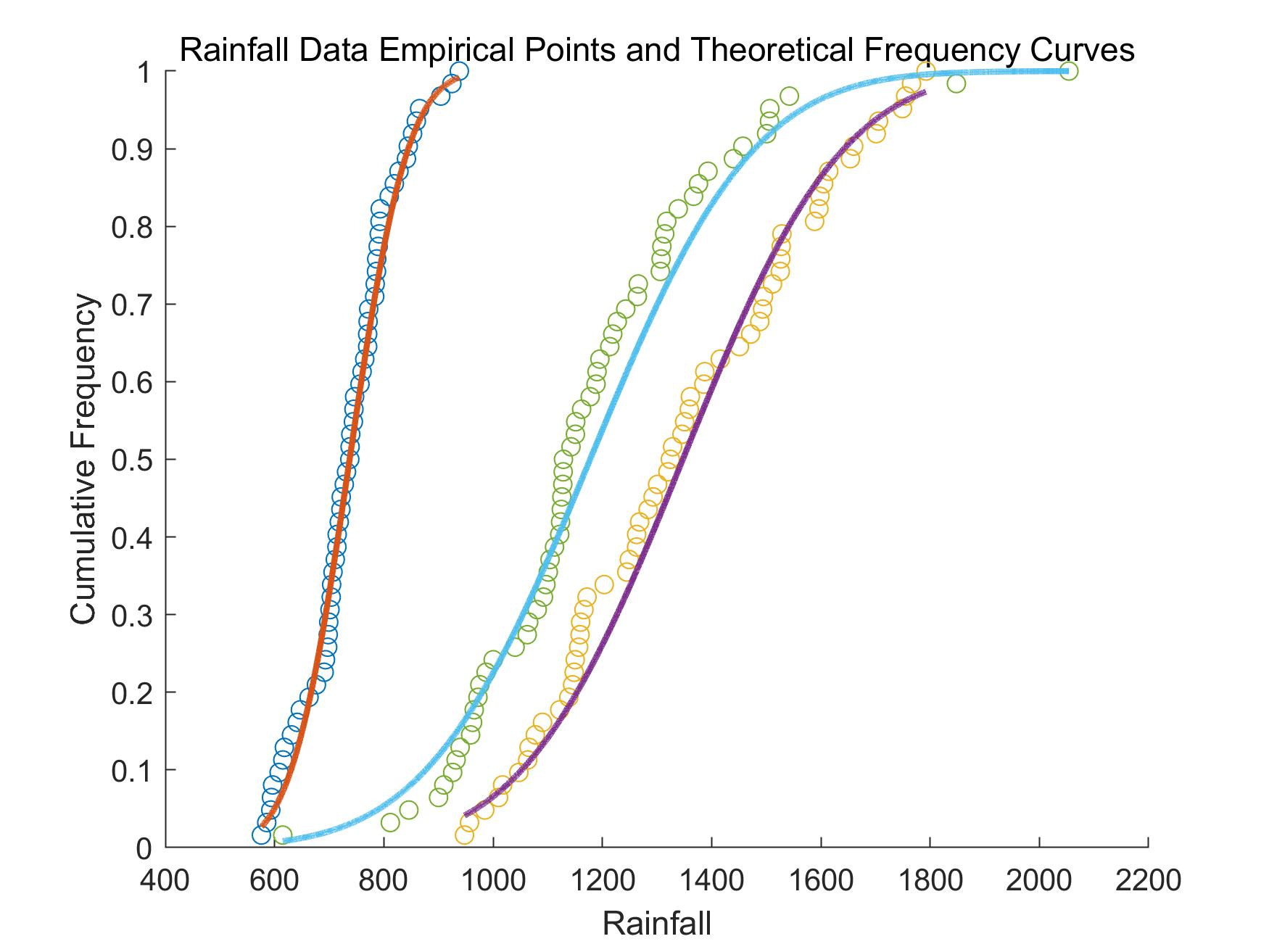
Fig A4. Empirical points and theoretical frequency curves of rainfall data during year-round.

Table A3. Parameter values of two-dimensional Copula functions

| The type of the function | Upstream-Midstream | | | Upstream-Downstream | | | Midstream-Downstream | | |
| --- | --- | --- | --- | --- | --- | --- | --- | --- | --- |
|  | Flood period | Dry period | Year round | Flood period | Dry period | Year round | Flood period | Dry period | Year round |
| Kendall τ | 0.23497 | 0.39234 | 0.2032 | 0.2437 | 0.3038 | 0.2360 | 0.3398 | 0.4502 | 0.4054 |
| Gaussian | 0.36077 | 0.57802 | 0.3139 | 0.3735 | 0.4593 | 0.3623 | 0.5088 | 0.6497 | 0.5947 |
| Frank | 1.13989 | 1.26967 | 1.1179 | 1.1461 | 1.1919 | 1.1406 | 1.2220 | 1.3288 | 1.2824 |
| Clayton | 0.61428 | 1.29136 | 0.5102 | 0.6445 | 0.8728 | 0.6180 | 1.0298 | 1.6381 | 1.3639 |
| Gumbel | 1.30714 | 1.64568 | 1.2551 | 1.3222 | 1.4364 | 1.3090 | 1.5149 | 1.8190 | 1.6819 |
| t | 0.37354 | 0.06103 | 0.0311 | 0.4384 | 0.4734 | 0.3944 | 0.5318 | 0.6770 | 0.0604 |

Table A4. Optimal fitting results of two-dimensional Copula functions" in an academic paper context in English

| Copula function | Basin | Flood period | | | Dry period | | | Year round | | |
| --- | --- | --- | --- | --- | --- | --- | --- | --- | --- | --- |
|  |  | K-S | OLS | RMSE | K-S | OLS | RMSE | K-S | OLS | RMSE |
| Gaussian | Upstream-Midstream | 0.8614 | 10.6342 | 0.4141 | 0.9030 | 7.7420 | 0.3534 | 0.0470 | 0.0604 | 0.0312 |
|  | Upstream-Downstream | 0.8159 | 9.3496 | 0.3883 | 0.0792 | 0.1513 | 0.0494 | 0.9001 | 7.1043 | 0.3385 |
|  | Midstream-Downstream | 0.0820 | 0.1566 | 0.0503 | 0.9357 | 8.3907 | 0.3679 | 0.8038 | 11.327 | 0.4274 |
| Frank | Upstream-Midstream | 0.7769 | 7.7800 | 0.3542 | 0.9248 | 10.5287 | 0.4121 | 0.0561 | 0.0762 | 0.0351 |
|  | Upstream-Downstream | 0.8352 | 10.9849 | 0.4209 | 0.0958 | 0.1907 | 0.0555 | 0.8273 | 10.178 | 0.4052 |
|  | Midstream-Downstream | 0.0937 | 0.1782 | 0.0536 | 0.9550 | 9.5407 | 0.3923 | 0.9005 | 11.182 | 0.4247 |
| Clayton | Upstream-Midstream | 0.8413 | 8.0006 | 0.3592 | 0.7274 | 9.2383 | 0.3860 | 0.0634 | 0.0861 | 0.0373 |
|  | Upstream-Downstream | 0.8634 | 8.1437 | 0.3624 | 0.0807 | 0.1433 | 0.0481 | 0.9056 | 7.5997 | 0.3501 |
|  | Midstream-Downstream | 0.0789 | 0.1320 | 0.0461 | 0.8803 | 11.2133 | 0.4253 | 0.8793 | 10.172 | 0.4050 |
| Gumbel | Upstream-Midstream | 0.7747 | 7.8912 | 0.3568 | 0.8843 | 9.5395 | 0.3923 | 0.0542 | 0.0703 | 0.0337 |
|  | Upstream-Downstream | 0.8295 | 8.8021 | 0.3768 | 0.0802 | 0.1544 | 0.0499 | 0.8598 | 9.1585 | 0.3843 |
|  | Midstream-Downstream | 0.0720 | 0.1102 | 0.0422 | 0.9653 | 12.2967 | 0.4453 | 0.8234 | 10.817 | 0.4177 |
| t | Upstream-Midstream | 0.8349 | 10.1343 | 0.4043 | 0.8829 | 9.8051 | 0.3977 | 0.0470 | 0.0608 | 0.0313 |
|  | Upstream-Downstream | 0.9655 | 11.1222 | 0.4235 | 0.0794 | 0.1520 | 0.0495 | 0.8582 | 9.6454 | 0.3944 |
|  | Midstream-Downstream | 0.0820 | 0.1566 | 0.0503 | 0.8336 | 7.4267 | 0.3461 | 0.8457 | 9.1105 | 0.3833 |

Table A5. Test for optimal fitting results of two-dimensional Copula functions

| Copula function | Flood period | | | Dry period | | | Year round | | |
| --- | --- | --- | --- | --- | --- | --- | --- | --- | --- |
|  | K-S | OLS | RMSE | K-S | OLS | RMSE | K-S | OLS | RMSE |
| GH | 0.0102 | -420.45 | 0.2806 | 0.12903 | -182.728 | 0.2743 | 0.0591 | -187.6 | 0.28256 |
| Clayton | 0.0752 | 555.761 | 0.2767 | 0.07526 | 224.242 | 0.2793 | 0.0430 | 224.97 | 0.27947 |
| Frank | 0.0591 | 614.982 | 0.2774 | 0.10215 | 245.319 | 0.2712 | 0.0860 | 246.58 | 0.27874 |


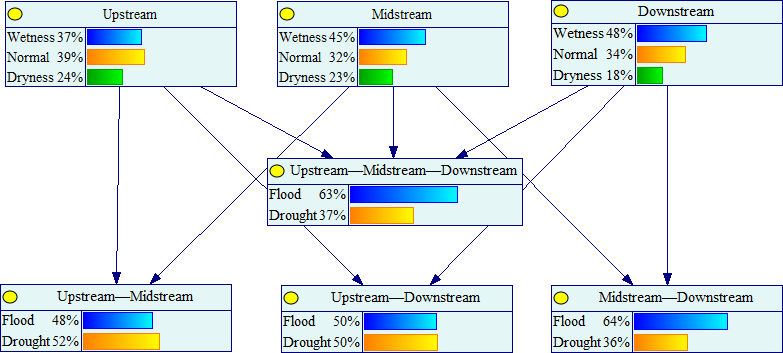
 Fig A5. Prior Inference Diagram of the Bayesian Network Model for Flood and Drought Management.


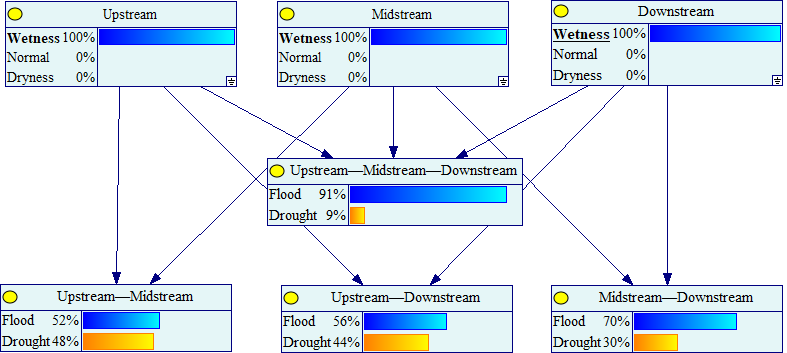


(a)


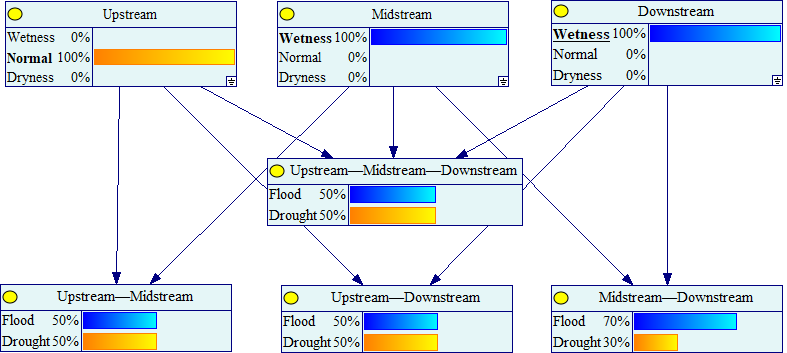


(b)

Fig A6. Simulation Results of Wet Year Scenarios using the Bayesian Network Model (a) and (b).


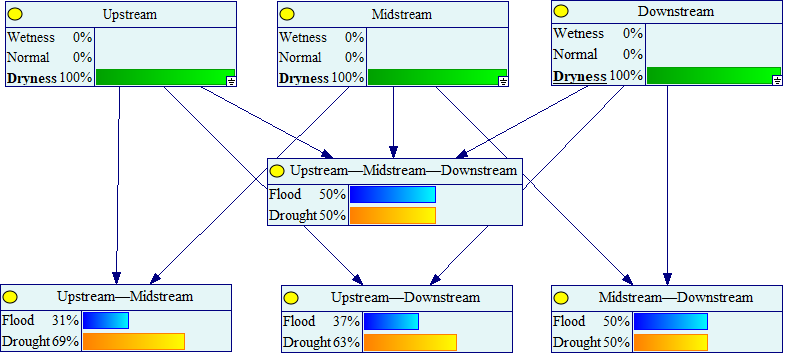


(a)


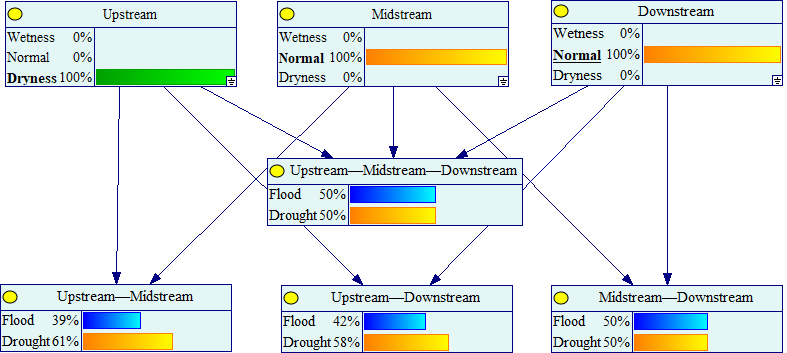


(b)

Fig A7. Simulation Results of Dry Year Scenarios using the Bayesian Network Model (a) and (b).
